# Characteristics, expectations, experiences of care, and satisfaction of patients receiving chiropractic care in a French University Hospital in Toulouse (France) over one year: a case study

**DOI:** 10.1186/s12891-022-05147-6

**Published:** 2022-03-09

**Authors:** Mallard F, Lemeunier N, Mior S, Pecourneau V, Côté P

**Affiliations:** 1 Division of Graduate Studies, Canadian Memorial Chiropractic College (CMCC), Toronto, Ontario Canada; 2 Institut Franco-Européen de Chiropraxie, Toulouse, France; 3 Faculty of Health Sciences, Ontario Tech University, Oshawa, Ontario Canada; 4 UMR1295, Toulouse III University, Inserm, Equipe EQUITY, Equipe constitutive du CERPOP, Toulouse, France; 5 Institute for Disability and Rehabilitation Research, Ontario Tech University, Oshawa, Ontario Canada

## Abstract

**Background:**

In October 2017, a partnership was established between the University Hospital of Toulouse and the French Chiropractic College, “Institut Franco-Européen de Chiropraxie” (IFEC). Before 2017, chiropractors did not practice in hospitals in France. Chiropractic students and chiropractors are now integrated in an interdisciplinary medical team at University Hospital.

Our study aimed to describe the characteristics of patients who received chiropractic care at the University Hospital of Toulouse, their expectations, experiences of care, and satisfaction.

**Method:**

A prospective case study was conducted. Patients referred for chiropractic care in the French University Hospital of Toulouse from January to December 2020 were eligible to participate. Participants provided the following data: demographics, previous chiropractic care treatments, pain location, intensity (NRS) and duration, disability (NDI, ODI), health-related quality of life (SF-12) and depressive symptomatology (PHQ-9). We conducted semi-structured interviews to explore their expectations, barriers and facilitators impacting their experience of care, and satisfaction.

**Results:**

Seventeen participants were recruited and seven were interviewed. All participants had chronic pain with a median pain intensity of 05/10 (IQR 04–06) on the NRS scale. Nine of 17 participants presented with multiple pain locations. Thirteen of seventeen participants presented with low back pain and eight with neck pain. The median SF-12 health-related quality of life score was 50/100 (IQR 28.5–60.5) for physical health, and 52/100 (IQR 43–62) for mental health. The PHQ-9 median score of depressive symptomatology was 7.7/27 (IQR 2.0–12.5).

Overall, participants were satisfied with their care and the collaboration between chiropractors and physicians. Participants expected a caring communication with the chiropractic team. Their experience was facilitated by their trust in their physician. Patients perceived the turnover of chiropractic students as a barrier to their satisfaction.

**Conclusion:**

Our participants presented with chronic musculoskeletal pain and depressive symptoms. Our study identified facilitators and barriers for patient expectation and satisfaction with chiropractic care in a hospital setting. This study provides the first data describing the collaboration between chiropractors and physicians in France in the management of musculoskeletal disorders. These findings will inform the quality improvement of our partnership, student’s training and the development of future hospital-based collaborations integrating chiropractic care in a multidisciplinary team in France.

**Supplementary Information:**

The online version contains supplementary material available at 10.1186/s12891-022-05147-6.

## Background

Chiropractic is a regulated health profession that focusses on the diagnosis, treatment, prognosis, and prevention of musculoskeletal conditions. In France, the only chiropractic college, the *Institut Franco-Européen de Chiropraxie* (IFEC) was established in 1983, but the profession was legalized in 2002 [1], became regulated in 2011, [2] and by decree in 2018 [3]. The regulations define the scope of practice of chiropractors, including granting the ability to diagnose and manage patients with musculoskeletal disorders without a medical referral. Despite legislative recognition, integration of chiropractors within the mainstream healthcare system has been slow and challenging in France. Successful models of integration exist in Canada [4], Denmark [5], and Switzerland [6] where chiropractors provide care in public hospitals and clinics. Developing similar models and partnerships culminating with the integration of the chiropractic profession into the mainstream health care system in France is a profession priority. A first step towards system integration started in 2017 with the development of clinical guidelines for patients with neck pain labelised by the *Haute Autorité de Santé* (H.A.S.) [7–12] an independent public health authority in France. The resultant recommendations support the French chiropractic profession’s commitment to delivery of evidence informed patient care consistent with expectations of a contributing member of the mainstream health care system.

Another example involves the first clinical partnership in France between the University Hospital in Toulouse, France, and IFEC, established in October 2017. This partnership involves chiropractic interns and clinicians delivering care within multidisciplinary teams that include medical doctors and interns, podiatrist, sport trainer, and nurses. The partnership was created to develop a multidisciplinary approach to the management of musculoskeletal disorders within a hospital environment. Similar approaches have been found to benefit patients, particularly those with chronic and complex conditions usually encountered in this environment [4]. Furthermore, this partnership was created to remove the economic barrier for patients of moderate to low income. Finally, the partnership provides chiropractic students with the opportunity to assess and manage patients, and to learn how to interact with other health care practitioners in a public hospital environment.

Since October 2017, 5^th^ and 6^th^ year undergraduate students from IFEC have been evaluating and managing hospital patients with musculoskeletal conditions under the supervision of a chiropractor in a multidisciplinary team. There are no other chiropractors currently working in the hospital. Chiropractic evaluation and management are available to patients on part-time schedule (1 day and a half per week). The initial visit is one hour long, and subsequent treatment visits are 30 min. On average, 12 new patients are seen per month, with a one month waiting list for an initial evaluation. Students evaluate patients with MSK disorders with frequent comorbidities such as traumatic, neoplasic, and rheumatoid disorders. Students are selected, trained, and attend to patients in the hospital once a week over a 6 to 8 weeks rotation. Patients are referred for a chiropractic evaluation on an outpatient basis, by health care professional from the sports medicine department of the hospital. Patients cannot access a chiropractic evaluation without a medical referral.

Following the evaluation, a decision to initiate chiropractic care is discussed at a round table meeting that involves the chiropractic students and supervising chiropractor, and medical doctors and interns. The discussion considers the need for special investigation, referral to another practitioner, goals of the care program (delivered either in-hospital or at the IFEC clinic), treatment modalities, and follow-up. The resultant clinical plan is summarized in a written report, including history and clinical examination findings, and management decisions, with consideration of patient preferences. A written consent form is signed by the patient and the chiropractor prior to initiating care.

Currently, little is known about the management of patients by chiropractors in a multidisciplinary environment in a French public hospital. Our primary objective was to describe the demographics and health characteristics of patients who received chiropractic care at the French University Hospital of Toulouse. Secondly, we aimed to describe whether patients’ expectations aligned with their experiences of the chiropractic care received at the Hospital, and the barriers and facilitators regarding their experience of care. Finally, we aimed to describe the satisfaction of our patients.

## Method

We used a prospective case study design. This design involves collecting data from a combination of different sources of evidence, using both quantitative and qualitative techniques [13]. Case studies are used to investigate a phenomenon from different perspectives to provide a deeper description and understanding within a bounded context [14].

### Study population

Adult patients from the “Occitany Region”, South West of France, presenting to a sport medicine department at the University Hospital in Toulouse city were recruited. Patients are referred for a chiropractic evaluation by health care professional from the sports medicine department of the hospital.

### Study sample

Inclusion criteria included patients who: 1) had an appointment for a chiropractic evaluation in the sport medicine department in the University Hospital of Toulouse city; 2) were ≥ 18 years of age; and 3) reported neck pain and associated disorders (including grade I, II, and III) or non-specific low back pain. Definitions of neck pain and low back pain are reported in Table 1.

**Table 1:** Definitions of neck pain and associated disorders [13], and non-specific low back pain [15]

Grade I neck associated disorders	No signs or symptoms suggestive of major structural pathology and no or minor interference with activities of daily living
Grade II neck associated disorders	No signs or symptoms of major structural pathology, but major interference with activities of daily living
Grade III neck associated disorders	No signs or symptoms of major structural pathology, but presence of neurologic signs such as decreased deep tendon reflexes, weakness, or sensory deficits
Non-specific low back pain	Non-specific low back pain is defined as low back pain not attributable to a recognisable, known specific pathology (e.g., infection, tumour, osteoporosis, fracture, structural deformity, inflammatory disorder, radicular syndrome, or cauda equina syndrome)

Exclusion criteria were patients with grade IV neck pain and associated disorders and/or low back pain attributed to a specific pathology (e.g., infection, neoplasm, metastasis, osteoporosis, inflammatory arthropathies, fractures).

## Phase 1: Method for the quantitative phase

### Recruitment

We recruited patients, at their initial visit, from January 2020 to December 2020 at the University Hospital of Toulouse. All patients referred for a chiropractic evaluation during this period were eligible for participation. Those eligible for participation were met in the waiting room and invited to a private study room to confirm their eligibility using a standardized checklist (inclusion and exclusion criteria) (Appendix B). If the patient was eligible, the chiropractor described the study objectives and invited the patient to enrol in the study. Participation was voluntary. Eligible patients were asked to provide written informed consent. Consenting patients then completed the study questionnaire.

### Data collection

We used a standardized electronic questionnaire to collect data. The questionnaire was administered face-to-face by the treating chiropractor. The questionnaire included questions about demographics (gender, age, occupation), pain (location, intensity, and duration), disability, health-related quality of life, and depressive symptomatology.

### Outcomes

#### Pain intensity

Neck and low back pain intensity were measured with the 10-point numerical rating scale (NRS). The NRS is a measure of pain intensity ranging from 0 (referring to "No pain") to 10 (referring to "Pain as bad as it could be"). The NRS is a reliable tool used in clinical practice to evaluate pain in the general population [10, 15]. Comparing with other pain scales, NRS is easier to use [16]. We classified our results as chronic, subacute, and acute pain. We defined pain lasting less that 6 weeks as acute, between 7 and 12 weeks as subacute, and pain ≥ 3 months as chronic [17].

#### Disability

Disability was measured using the Oswestry Disability Index (ODI) for low back pain and Neck Disability Index (NDI) for patients with neck pain. A score of 0 represents no disability and 100 represents the debilitating disability. Both are reported to be reliable and valid measures for neck and back-related disability [17, 18]. ODI test–retest reliability is ICC = 0.83 in patients with low back pain [18]. The NDI test–retest reliability is ICC = O.91 (95% CI 0.83–0.96) in adults with NAD I–III of unspecified duration and a good construct validity in patients with neck pain [19]. The transcultural evaluation in French has also been validated for both questionnaires [20, 21].

#### Health-related quality of life (H-RQoL)

We used the Medical Outcomes Study Short-Form Health Survey version two (SF-12) to measure health-related quality of life (H-RQoL). The SF-12 has 12 items that measure the H-RQoL of a subject. SF-12 is divided into two subscales: physical component summary (PCS-12) and mental component summary (MCS-12). Each score is calculated over 100. Higher scores reflect better quality of life. It has been shown to be a good alternative to SF-36. In the general population, the short form 12-item survey demonstrated good test–retest reliability [22] and good internal consistency with a Cronbach alpha coefficient of 0.77 for PCS-12 and 0.80 for MCS-12 [23]. The transcultural evaluation in French has also been validated [24]. Studies in France revealed a median score of 54.1 for PCS and 51.7 for MCS in the French population [25].

#### Depressive symptomatology

Depressive symptomatology was measured with the Patient Health Questionnaire (PHQ9). It consists of nine questions designed to correspond to the nine diagnostic criteria for major depressive disorder. Each of the 9 items are rated from 0 to 3. Final score can range from 0 to 27. The score can then be interpreted as indicating either no depression, mild (score 5–9/27), moderate (score 10–14/27), moderately severe (score 15–19/27), or severe depression (score ≥ 20/27). It has shown to have high internal consistency with a Cronbach alpha coefficient of 0.83 and a high test–retest reliability in the general population [26, 27]. The transcultural evaluation in French has also been validated [28].

#### Analysis

For categorical variables (gender, location of pain, duration of pain, previous chiropractic treatment), we calculated counts and percentage with their 95% confidence intervals. For continuous variables, we computed median and quartiles.

### Phase 2: Method for the qualitative phase

#### Recruitment

All participants in phase 1 were invited to participate in phase 2. We purposively recruited participants from our initial phase 1 sample. Participants were sequentially contacted by phone by an additional external chiropractor of the hospital and invited to participate. This external chiropractor provided details about the study and answered questions by phone. If the participants agreed, an interview appointment was made at mutually convenient day and time. Interviews were conducted over a one-month period.

#### Outcomes

During the interviews, we gathered information to explore the participant’s satisfaction, expectation, and experiences with care. Satisfaction has been defined as “positive evaluations of distinct dimensions of healthcare”[29]. Patient’s expectation can be defined in terms of needs, request of desires prior to seeking the doctor [30]. Different definitions of patient experience exist. It can be defined as ‘feedback from patients on what actually happened in the course of receiving care or treatment, both the objective facts and their subjective view of it’. The experience may be influenced by patient diversity and may be affected by their previous experiences with care. Understanding and considering patient’s experience will help to achieve excellence in care. Thus, gathering the barriers and facilitators impacting the experience of care may improve quality and outcomes by considering the patient at the center of the decision making [31].

#### Data collection

All interviews were conducted by phone. The principal investigator (PI), a chiropractor, was trained by an experienced qualitative researcher and conducted all the semi-structured interviews. The interviews were guided by a list of open-ended questions, including related probes (Appendix A). Saturation may be achieved with as few as 6 to 8 patients or up to 12 to 30 patients when looking for disconfirming evidence or trying to achieve maximum variation [32, 33]. Interviews were audio recorded and transcribed verbatim. The PI reviewed the transcribed interviews to ensure their accuracy. The PI then contacted participants if they wanted to review their transcript, and edit their responses (member checking). Patients were able to modify their transcripts and/or withdraw from the study.

#### Analysis

We used qualitative content analysis to code transcripts [34]. Each transcript was read and coded independently by two members of the research team. Open coding of passages was used, and codes then sorted into independent categories using an inductive approach [35]. The two members then met to discuss differences and reach consensus of the generated codes and categories. Once consensus was reached, qualitative data were analysed in combination with the quantitative part to understand patients’ expectation, satisfaction, and experience of care. Facilitators and barriers impacting their experience of care were also identified and analysed.

#### Ethics

This study was approved by Canadian Memorial Chiropractic College (CMCC) ethics committee (REB #1906B03) and Personal Protection Committee in France (CPP Ile de France X) (RC31/19/0323 2019-A02176-51).

## Results

### Quantitative findings

#### Demographics characteristics and previous chiropractic experience

Table 2 describes the participant demographic characteristics. Seventeen participants were invited to participate. All of them were enrolled, of which 11 were female, most were between 41 and 70 years of age and employed. This low number of recruited participants is associated with the Covid crisis that resulted in the restricted access to chiropractic services between March 2020 and September 2020. In a normal period, we should have potentially recruited more than 100 participants.

**Table 2:** Demographic characteristics of the sample

Variables		n (%)
Gender	Women	11 (65)
	Men	6 (35)
Age Category	18–40	7 (41.2)
	41–70	10 (58.8)
Occupation	Employed	7 (41.3)
	Unemployed	10 (59.7)

#### Health characteristics

Nine of 17 participants presented with multiple pain locations, primarily localized to low back with or without lower extremity pain. The second main clinical presentation was neck pain with or without headache or upper extremity pain (Table 3). Half of our participants had never seen a chiropractor before.

**Table 3:** Pain locations of participants

Pain location	Participants (n)
Participants with low back pain with or without lower extremity pain	13
Participants with neck pain with or without headache or upper extremity pain	8
Participants with back pain	4

Thirteen of seventeen participants completed the low back pain questionnaire. Most participants presented with minimal disability, with a median ODI of 16/50 (IQR 12–38). One ODI questionnaire was incomplete. All 13 participants had chronic pain with a median intensity of 5.5/10 (IQR 4–6). Seven of seventeen participants completed the neck pain questionnaire. All 7 participants had chronic pain with a median pain intensity of 6/10 (IQR 4–6).

All our participants completed the PHQ9 for depression and SF12 for quality of life. Regarding the PHQ9, eight participants were classified as none-minimal, one as mild, five as moderate and three as moderately severe for severity of depression. The median PHQ9 was 7.7/27 (IQR 2–12.5). The results of the SF12 highlighted a median score of 50/100 (IQR 28.5–60.5) for physical health scale and 52/100 (IQR 43–62) for mental health scale (Table 4).

**Table 4:** Health characteristics of participants

Health characteristic variables	Median (IQR)	Category	N (%)
LBP intensity (NRS)	5.5/10 (4–6)	Chronic	12 (92.3)
		Subacute	1 (7.7)
Neck Pain intensity (NRS)	6/10 (4–6)	Chronic	7 (100)
LBP Disability (ODI)	16/50 (12–38)	Minimal disability	n = 7 (53.8)
		Moderate to severe disability	n = 5 (38.5)
		Incomplete	n = 1 (7.7)
NAD disability (NDI)	18/50 (8.5–28.5)	Mild to Moderate disability	n = 5 (62.5)
		Severe disability	n = 3 (37.5)
Depression (PHQ9)	7.7/27 (2–12.5)	None-minimal to Mild	n = 9 (52.9)
		Moderate to moderately severe	n = 8 (47.1)
Health related quality of life (SF-12), Physical Health Scale	50/100 (28.5/100–60.5/100)		
Health related quality of life (SF-12), Mental Health Scale	52/100 (43/100–62/100)		

#### Qualitative findings

Seven of seventeen participants were interviewed. Interviews were conducted until saturation of key themes was achieved. We explored the expectation, satisfaction, and experience of our participants, as well as the barriers and facilitators of the care they received. Emerging from each theme were related categories detailing a more in-depth perspective shared by participants.

#### Expectation of chiropractic care

The most prevalent expectation reported by the participants was relief of their pain. Pain relief appeared to be more important than improvement in their physical functioning. However, this perception differed depending on the chronicity of the participant’s symptoms, where those with higher levels of self-reported disability expected more improvement in their overall condition rather than pain relief. Participants with higher levels of disability appeared accepting of their pain, appreciating that a “cure” was unlikely but expecting improvement in their ability to function. They were able to differentiate satisfaction with the care as linked to functional outcomes compared to those in acute pain who considered pain relief. As highlighted by a chronic participant, *“My main expectation was really …. I knew that total healing no, but relief, yes.”* (Pt#3).

Other participants were uncertain of their expectation with chiropractic care. This uncertainty related to their lack of prior experience with such care, resulting in a sense of ambivalence or apprehension. The apprehension was due to their perception of chiropractic care, which usually involves manipulation, envisioning it as more aggressive than other forms of conservative care. For example, *“what reflects chiropractic, how to say, it's the opposite of gentle. It's the manipulation that is a little brutal.”* (Pt#2). Regardless of the underlying reason for their uncertainty, these participants relied on their trust with their physician making the referral. This trust appeared to enable them to attend for care, be open to the outcomes of care, and countered their apprehension, as noted by participant #5:

“I said to myself well, here is another new thing that I can test and then we will see. I was neutral, I did not know. I was not enthusiastic (laughs).”

One common expectation among all participants was the perception of empathy. They expected an empathetic provider who listened and communicated with them in a humanly, caring and understanding manner. They appreciated being spoken to, rather than of, during their interaction. Such communication was not limited to conveying the treatment plan but also providing reassurance about the nature of their condition. As highlighted by one patient, this caring communication significantly impacted not only their quality of life but also their depressive symptoms:

*“For me, above all, the care also depends on the character* (of the doctor) *....one who is very human, who dialogues a lot with his patients […] having a doctor who is listening, who is patient… already that reassures* (me)*.”* (Pt#3)

#### Satisfaction of chiropractic care

Most participants were satisfied with the chiropractic care they received at the hospital and would recommend it for different reasons. As highlighted by a participant that *“In terms of efficiency, for me it was quite spectacular, it did me a lot of good, so I highly recommend it.”* (Pt#4). However, most participants did not consider that complete healing was a prerequisite for satisfaction. On the contrary, participants highlighted that a caring communication was a higher source of satisfaction to them. They reported that they were satisfied by the caring and empathetic communication between them and the chiropractic team. Participants expected such communication, and achieving this expectation was for them a source of satisfaction. This caring communication and its impact were highlighted by a participant with chronic pain:

*“I am very satisfied with the chiropractic team […], they have a very human side.”* (Pt#3)

The collaboration between the chiropractors and the medical team was also reported as a source of satisfaction by our participants. They highlighted the different aspects regarding this collaboration, such as the organisation of the care, the impact of the discussion of clinical case, and the exchange of information between the chiropractic and the medical teams. Awareness of communication between the two teams was highlighted as an important factor contributing to their satisfaction. This was specifically highlighted by participants with higher levels of depression and disability. They were specifically satisfied that the team discussed their clinical case and was aware of their clinical file and condition. Our participants expressed that it helped that the team understood their past medical history. Most of our participants expressed the impact of this communication on their expectation and outcomes. As highlighted by a participant with low back pain:

“If we feel that the team is informed on our file, we will be more attentive, less reluctant.” (Pt#7).

Finally, the organisation and supervision of the care was also described as a source of participants’ satisfaction. They explained that they enjoyed being part of the teaching environment, knowing that this was beneficial to the chiropractic students and important in helping to develop their skills. They also described that they particularly enjoyed the supervision of the chiropractic students by the chiropractor. One patient highlighted that *« This is very professional and well supervised» (Pt#9).*

#### Experience of chiropractic care

The experience of care of our participants was explored. This helped us to understand the facilitators and barriers impacting the care they received.

The most frequently mentioned facilitator reported by our participants was their trust in the medical team who referred them to the chiropractor. This was especially important to those participants who did not have any previous experience with chiropractic care or who had uncertain expectations. Those participants highlighted that the referral made by the physician had an important impact on their decision to receive a chiropractic care, as noted one chronic participant: *“It was important that it was a doctor who advised you? “Yes, that proves, in a way, a certain value behind.” (Pt#7).* Without this referral, some participants expressed that they would not have visited the chiropractor. This trust appeared to enable them to attend for care, as noted by participant #7:

“I might not have gone to see a chiropractor like this on my own. The fact that he's a doctor [who sent me] for me is important.”

This trust in the medical team also enabled participants to recognize the chiropractor as an expert in the musculoskeletal field, even if they were apprehensive. Knowing that the referral was made by a physician reassured the patient. This facilitator was mentioned by participant #4:

*“I think the doctor could have referred me to the specialists who were best suited to treat my condition. Because he may not have the skills in chiropractic, so he sent me to you ... it may have helped me better heal.”*

Our participants’ experience was positively influenced by the location of the chiropractic care in the Hospital environment. This co-location of care was seen by the participants as a facilitator of communication between the medical and chiropractic team. This co-location helped our participants quickly access care and improved the exchange of information and communication between the medical and the chiropractic teams. This was described as a source of satisfaction by one of our participants:

“Since the chiropractors' offices are right next door, they were going to make a quick point with the chiropractors. And they saw each other a little more to talk about my case in fact.” (Pt#4)

There were also noted barriers to chiropractic care. First, the participants’ attitude toward chiropractic care was perceived as an important barrier. Some participants were apprehensive or uncertain regarding chiropractic treatment. The negative perception of their families towards chiropractic reinforced this feeling of apprehension. One participant highlighted: *“around me, people did not approach it in a positive way, there is always the fear of manipulation and the consequences of manipulation” (Pt#4).* Especially for these participants, the trust in the medical doctor who referred them was essential to balance this barrier.

Even if the communication between the chiropractic team was highlighted as a facilitator, some participants were more reluctant. Some expressed that they did not really know if the team communicated well. They were not aware of the multidisciplinary meetings organised between the chiropractic and the medical teams to discuss their management. One participant highlighted:

*“I don't have the impression that there are meeting to discuss the [management of] patients. There should be more communication between them, that's for sure. Because I don't feel like there is.“ (Pt#2)*

Finally, some participants noted dissatisfaction and apprehension being treated by chiropractic students. Indeed, our 6^th^ year undergraduate students manage patients in the hospital setting on a 6-weeks rotation. This can lead to challenges regarding the continuity of patient’s care. The chiropractor is responsible for maintaining the continuity of care by ensuring the transition between each pair of students. However, participants noted that they had to repeat the same information during each new turnover, intimating a sense of frustration. Some chronic participants highlighted that this 6-weeks turnover was too frequent.

“Where it is painful, it is that we consult with new students so each time we start from scratch somewhere, because the team changes often and so we start again.” (Pt#2)

Moreover, some participants also highlighted the lack of experience of some of the students. Students are prepared and trained before treating patients in hospital. However, patients from the chiropractic school clinic may differed from those at the hospital. The student’s lack of experience was described as a source of dissatisfaction by our participants.

*« Afterwards I didn't tell them I was reluctant about what ... They did their manipulation and after I felt that they weren't too sure of themselves, but I didn't tell them anything, he didn't notice it that I was a little reluctant.” (Pt#7)*

## Discussion

To our knowledge, this is the first prospective case study describing the chiropractic management of patients in a public hospital environment in France. Our findings are similar to previous studies describing chiropractic patients, who most commonly present with low back pain and neck pain, with or without extremity pain [36]. All our participants had chronic pain and most presented with multiple complaints. Facing complex and chronic clinical cases is fundamental for chiropractic student’s training. Previous studies in chiropractic college clinics suggested that chronic/recurrent conditions were twice less likely to be reported as acute complaints and considered uncomplicated [37].

Our study helped us to understand participant’s expectations, considering that patient’s expectation can maximize patient’s outcomes [38]. Understanding, exploring, and meeting these expectations are part of patient-centered care approach. Recognizing patient’s preferences, choices, and experience are key factors in patient management of musculoskeletal complaints [39]. It helps to achieve excellence in care in a patient centered approach [40]. Exploring their experiences and their expectation also help to improve the shared decision-making process and reinforces the therapeutic alliance [41]. In our study, some participants were unsure of their expectations about their prognosis and management but presented with specific expectations regarding their pain and their disability. Most participants had high expectations regarding a manipulative approach for their management. This is similar with other studies describing the expectations of patients with neck and low back pain [42–44]. Our participants appeared to present with specific expectations. This was especially noted with participants with high levels of disability, depressive symptomatology, or decreased quality of life. Their expectations were lower than participants with lower levels of disability. However, they did expect a caring and empathetic communication and a good relationship with the medical and the chiropractic team.

Our participants expected relief of pain and an empathetic and caring communication with the medical and chiropractic team. This caring communication impacted participant’s level of pain and disability. Evidence suggests a positive association between clinical empathy and improved therapeutic outcomes. Clinical empathy is an important component of the physician–patient therapeutic relationship [45, 46]. One patient reported an impact of this communication on his quality of life and depressive symptoms. Regarding participant’s quality of life, findings from our small sample revealed a median score of 50/100 (IQR 28.5–60.5) for physical health scale and of 52/100 (IQR 43–62) for mental health scale. This is consistent with the French adult norm with a median score of respectively 54.1/100 for PHS and 51.7/100 for MHS [24]. Almost 50% of participants referred for chiropractic care in our study had moderate to severe depression. Although we lack information about PHQ-9 scores in the general population in France these findings are important to guide the training of the IFEC chiropractic students to ensure they are able to assess and identify depressive symptoms and know how to co-manage such conditions.

Participants with no prior chiropractic experience were apprehensive or had uncertain expectations. The trust in their medical doctors who referred them to chiropractors was essential and was recognized as a major facilitator of care. The importance of the trust between patients and their physicians in facilitating access to complementary and alternative medicine, including chiropractic care has been highlighted in previous studies [14, 47]. It is not surprising that half of our participants had no prior experience with chiropractic care because the chiropractic profession is in development and relatively new in France. However, we lack information about the utilization rate of chiropractic services in France to reflect on our findings.

We identified other facilitators of care through our interviews. The co-location of care and communication between the chiropractic and physician teams were noted by the participants as important. This finding is consistent with previous studies suggesting co-location facilitates communication between collaborative care providers [48, 49]. Multi-disciplinary co-location is related to positive outcomes at a physician level, especially increasing collaboration among different providers and wider coordination with secondary care [48]. Co-location is likely to enhance communication and teamwork, resulting in more efficient care for patients. This can lead to clearer and faster communication between care team members [50]. This may also positively influence interprofessional collaboration and trust between health care providers and patients [51].

Participant’s satisfaction was also explored in our study. Participants were satisfied by the care they received. They highlighted different sources for their satisfaction: 1) caring communication by the chiropractic team, 2) the collaboration between chiropractor and physician, and 3) the organisation of the partnership. Despite the lack of improvement of care on pain or disability, they were satisfied by the caring communication they received from the chiropractic team. Communication quality has been shown in previous study to be a consistent predictor of patient satisfaction with chiropractic treatment [52].

Finally, our study identified barriers that may impact the experience of care received. Participants’ apprehensive attitude of chiropractic care was improved by the trust in the referring medical doctor. The lack of communication between the chiropractic team and the physicians was also considered a barrier to satisfaction that needs to be addressed. Indeed, interprofessional meetings between the medical and chiropractic team are organised weekly but participants were not aware. Finally, the turnover between the chiropractic students was identified as a barrier to satisfaction by participants. Frequent turnover appeared to negatively impact the continuity of care and frustrate participants. These barriers (apprehension) are similar with previous studies about the implementation of chiropractic care in a multidisciplinary environment [47, 53]. These barriers need to be understood and addressed in order to improve our partnership and meet our patient’s expectations. We will consider increase the duration of each turnover.

### Strengths and limitations

This study has strengths. We selected every patient coming for a chiropractic evaluation during a 5- month period in an hospital environment. We used a case study design to gather in-depth information about our sample. We used valid and reliable tools to describe our patients’ characteristics and qualitative semi-structured interviews to explore their experiences receiving a chiropractic care.

Our study has also some limitations. Our sample size is small because of the COVID-19 crisis and the resultant restricted access to the hospital for musculoskeletal care. We may also have social desirability bias, the tendency of patients to answer questions in a way that will be viewed and considered favorable by others, thus influencing responses. However, multiple probes were used to confirm their experiences. Confirmation bias may have occurred if the researcher interpreted responses to confirm their own belief [54]. However, we limited such bias by the researchers bracketing themselves during the interview process, and using self-reflection during debriefs post interview and analysis to avoid introducing personal bias [54]. The process also included two independent reviewers during the coding process to increase the validity and minimize bias. Finally, the COVID pandemic impacted our patient numbers because of several lockdowns in France, resulting in a decrease in the number of referrals and a pause in the partnership.

## Conclusion

Most participants presented with chronic neck and low back pain and depressive symptoms. We identified facilitators and barriers for patient expectation and satisfaction with chiropractic care in a hospital setting. These will need to be addressed in order to improve our partnership and the satisfaction of our patients. Future study should explore the practitioner’s experience and perspective.

This study provides the first data regarding the collaboration between chiropractors and physicians in France. These findings will inform the improvement of our partnership, student’s training and the development of future hospital-based collaborations integrating chiropractic care in a multidisciplinary team. 

## Supplementary Information

**Additional file 1:** **Appendix A:** Key informant interview guide. **Appendix B:** Standardized checklist for admission

## Data Availability

The data that support the findings of this study are available on request from the corresponding author [FM]. Ethics approval and consent to participate. This study was approved by Canadian Memorial Chiropractic College (CMCC) ethics committee (REB#1906B03) and Personal Protection Committee in France (CPP Ile de France X) (RC31/19/0323 2019-A02176-51). All methods were carried out in accordance with relevant guidelines and regulations. Participation was voluntary. Eligible patients were asked to provide written informed consent.
