# Supplementary material for: Characteristics, expectations, experiences of care, and satisfaction of patients receiving chiropractic care in a French University Hospital in Toulouse (France) over one year: a case study
Source: BMC Musculoskelet Disord. 2022 Mar 9;23:229. doi: 10.1186/s12891-022-05147-6 (PMC8906111; doi:10.1186/s12891-022-05147-6)
Supplement: Supplementary file 1 — Additional file 1: Appendix A: Key informant interview guide. Appendix B: Standardized checklist for admission [file 12891_2022_5147_MOESM1_ESM.docx]

Appendix A: Key informant interview guide

1. How long have you been under chiropractic care at the Toulouse University Hospital?
2. Did you see a chiropractor before? In not, what were your first thoughts when chiropractic care was suggested for you in this Hospital?
3. What did you first expect when you saw the chiropractor and chiropractic students?
4. Can you describe your experience with the chiropractic students? And with the chiropractor?
5. What role did you have in the plan of management?
6. Can you describe an average treatment you receive?
7. How satisfied were you with the care you received?
8. Did your experience with care meet your expectations?
9. Did your expectations change over time and why?
10. How do you perceive the collaboration between the chiropractic team and the rest of the health care team of the Toulouse University Hospital? What role you think this collaboration may play with your care?
11. Would you recommend for a patient to see chiropractic students and chiropractor in the Toulouse University Hospital for a care?
12. Any other comments that you wish to discuss?

Appendix B: Standardized checklist for admission

- Inclusion criteria:

1) have an appointment for a chiropractic evaluation in the sports medicine department at Toulouse University Hospital;

2) adults (≥18 years old)

3) have mechanical neck pain (including grade I, II, III neck pain) and / or mechanical nonspecific low back pain.

4) Be covered by a social security plan

5) Gave consent to the research project

- Non-inclusion criteria:

1. Patients with grade IV neck pain and / or low back pain secondary to a specific pathology (e.g. infection, primary tumor, secondary metastasis, osteoporosis, inflammatory rheumatism, fractures, ...).

2) Pregnant and / or lactating woman

3) Patients under legal protection, or guardianship, or curatorship
